# Follow-up care for men with prostate cancer and the role of primary care: a systematic review of international guidelines

**DOI:** 10.1038/sj.bjc.6605080

**Published:** 2009-05-12

**Authors:** H M McIntosh, R D Neal, P Rose, E Watson, C Wilkinson, D Weller, C Campbell

**Affiliations:** 1 Community Health Sciences – General Practice, University of Edinburgh, 20 West Richmond Street, Edinburgh EH9 9DX, UK; 2 Department of Primary Care and Public Health, North Wales Clinical School, Cardiff University, Gwenfro Unit 5, Wrexham Technology Park, Wrexham LL13 7YP, UK; 3 Deptartment Primary Care, University of Oxford, Old Road Campus, Headington, Oxford OX3 7LF, UK; 4 School of Health and Social Care, Oxford Brookes University, Jack Straws Lane, Marston, Oxford OX3 0FL, UK

**Keywords:** prostate cancer, follow-up, guidelines, systematic review

## Abstract

The optimal role for primary care in providing follow-up for men with prostate cancer is uncertain. A systematic review of international guidelines was undertaken to help identify key elements of existing models of follow-up care to establish a theoretical basis for evaluating future complex interventions. Many guidelines provide insufficient information to judge the reliability of the recommendations. Although the PSA test remains the cornerstone of follow-up, the diversity of recommendations on the provision of follow-up care reflects the current lack of research evidence on which to base firm conclusions. The review highlights the importance of transparent guideline development procedures and the need for robust primary research to inform future evidence-based models of follow-up care for men with prostate cancer.

Incidence rates of prostate cancer are rising in many countries, including the United Kingdom. It is now the most commonly diagnosed cancer in men in Europe (Ferlay *et al*, 2007) and the second most common male cancer worldwide (http://info.cancerresearchuk.org/cancerstats/types/prostate/incidence/). The numbers of men living with a diagnosis of prostate cancer will continue to increase as the population ages, and cancer is detected earlier with the more widespread use of prostate-specific antigen (PSA) testing.

Management options for localised and locally advanced prostate cancer include curative treatment, active surveillance and watchful waiting. The main curative treatments are radical prostatectomy, external beam radiotherapy (EBRT) and permanent (low-dose) brachytherapy. Hormone therapy (surgical or medical) may also be given as neoadjuvant, adjuvant or a stand-alone treatment for non-metastatic disease. Active surveillance is an option for men with low- or intermediate-risk localised disease that involves close monitoring to target curative treatment to those who would benefit most. Watchful waiting is a way to manage men who are unsuitable for curative treatment that involves relatively lax monitoring and palliative treatment when symptoms develop (NICE, 2008). Metastatic disease is treated palliatively with hormone therapy. Monitoring and post-treatment follow-up strategies aim to detect disease progression or recurrence, and manage long-term complications and treatment-related morbidity.

Traditionally, follow-up care has been hospital based and provided by clinical specialists in urology and oncology outpatient clinics. In practice, follow-up is provided in both primary and secondary care, and is not always well coordinated (Campbell *et al*, 2002; Neal, 2008) – this is despite widespread calls for better integrated care (Grunfeld, 2006; Oeffinger and McCabe, 2006; Department of Health, 2007; Nord *et al*, 2007). Further, there is evidence that prostate cancer patients are more likely to have a worse experience of care, including after care, than those with other cancers (Department of Health, 2005).

Various alternative models of cancer follow-up care have emerged, including nurse specialist and primary-care-led follow-up, and ‘shared care’ approaches. Only specialist nurse-led prostate cancer follow-up has been evaluated in randomised trials, which found it to be a safe alternative to consultant-led follow-up (Helgesen *et al*, 2000; Faithfull *et al*, 2001).

In the context of current widespread interest in greater involvement of primary care in cancer follow-up (Pascoe *et al*, 2004), we reviewed existing guidelines on follow-up for prostate cancer as part of a larger study to determine the optimal role for primary care. It is timely to review international guidelines to help (1) identify key elements of existing models of care and (2) establish a theoretical basis for evaluating future complex interventions.

## Materials and methods

### Search and selection

One reviewer performed the search and selection and a second reviewer verified the decisions on inclusion. Relevant guidelines were identified using the internet search engine SUMSearch, University of Texas, San Antonio, TX, USA (Hasse *et al*, 2007), followed by electronic searching of the individual websites of guideline collections (including clearinghouses and specialist libraries), guideline development agencies and professional societies, and finally the bibliographic databases MEDLINE, Bethesda, MD, USA and EMBASE (Elsevier, Amsterdam, The Netherlands). The searches were conducted from September to December 2007 with no language restriction. The sources searched are listed in Supplementary Table 1. References in the guidelines identified and personal contacts were consulted to identify additional guidelines. The pre-defined inclusion criteria were (1) the guideline was developed by a professional society or a national, regional, state or provincial government agency, (2) the guideline originated in the United Kingdom, Western Europe, Australia, Canada or the United States, (3) the guideline contained recommendations on post-treatment follow-up, active surveillance or watchful waiting, (4) the target group was primary or secondary health-care providers and (5) the date of issue was from 1990 onwards. Selection was thereby limited to current non-commercial guidelines and countries where the incidence of prostate cancer has risen over the past two decades and treatment options are similar.

Where necessary, the body that issued a potentially eligible guideline was contacted to obtain the full report. The most recent available version of updated guidelines was used. Additional reports and supporting material (such as journal articles and web pages) describing the guideline content, methods, development process or dissemination strategy, and tools for implementation were assembled for each included guideline.

### Data extraction

One reviewer extracted data using a pro-forma that was checked by a second reviewer if the guideline was published in English. Data extraction from guidelines not published in English was based on translations. Data were extracted for the recommendations on follow-up (including strategies for active surveillance and watchful waiting as well as post-treatment follow-up), sources of evidence, criteria used to grade the quality of the evidence and strength of the recommendations, and the composition of the guideline development group.

### Quality assessment

The Appraisal of Guidelines Research and Evaluation instrument was used to assess the quality of the included guidelines (www.agreecollaboration.org). It has 23 items in six domains: scope and purpose, stakeholder involvement, rigour of development, clarity and presentation, applicability and editorial independence. Assessing a guideline involves assigning each item a score of 1 (low) to 4 (high) and calculating a composite score for each domain. Domain scores are not aggregated. Two reviewers independently assessed the guidelines published in English. Assessment of foreign language guidelines was based on translations, supporting materials available in English and direct appraisal by native speakers to obtain, as far as possible, two independent appraisals.

## Results

### Included guidelines

Forty-one potentially eligible guidelines were identified. Eighteen met the inclusion criteria – these are described in Table 1 and are marked with an asterisk in the reference list. One was still in preparation. The 22 excluded guidelines are listed in Supplementary Table 2. The included guidelines were published between 1999 and 2008: 11 originated in Europe (three in the United Kingdom), three in the United States and four in Canada; 11 were produced by professional societies and seven by government agencies; and 13 were published in English, two in French and one each in Dutch, Finnish and Swedish. The scope of most of the guidelines is prostate cancer management (COIN, 1999; BCCA, 2001; ESMO, 2006; CCNS, 2006; ACB, 2007; CBO, 2007; EAU, 2007; FCCG, 2007; NCCN, 2007; SBHW, 2007; NICE, 2008); one is specific to follow-up (AFU, 2005); one is restricted to management of non-metastatic disease (SOR, 2006); and two are specific to permanent brachytherapy for localised disease (ESTRO, 2000; ACR, 2005). One guideline (OMHLTC, 2002) and one best practice policy statement (AUA, 2000) on PSA, and a guidance document on urological cancer (including prostate cancer) services (NICE, 2002) were also included.

### Guideline quality

Of the 18 guidelines reviewed, only the recent UK (NICE, 2008), Dutch (CBO, 2007) and Finnish (FCCG, 2007) guidelines, and the UK urological cancer services guidance (NICE, 2002), are of high overall quality according to the AGREE Collaboration's rating scheme, indicating that they could be considered for use in practice without provisos or alterations. The quality of the other 14 guidelines was either moderate (COIN, 1999; AUA, 2000; OMHLTC, 2002; ACR, 2005; CCNS, 2006; ESMO, 2006; SOR, 2006; SBHW, 2007) or low (ESTRO, 2000; BCCA, 2001; AFU, 2005; ACB, 2007; EAU, 2007; NCCN, 2007). The Collaboration considers low-quality guidelines to be more likely to have serious shortcomings and, therefore, not recommended for use in practice. The domain scores for each guideline are shown in Supplementary Table 3.

Most (14 out of 18) guidelines failed to describe their scope and purpose adequately, omitting details of the patient population to whom the guideline applied, the expected health benefits and the clinical questions they addressed. Six appeared to have been developed exclusively by clinical specialists (ESTRO, 2000; NICE, 2002; AFU, 2005; ESMO, 2006; SOR, 2006; EAU, 2007) and nine by multidisciplinary groups that also included other professionals from primary care (a GP or a specialist in family practice), nursing, psychology and social care (COIN, 1999; AUA, 2000; OMHLTC, 2002; CCNS, 2006; CBO, 2007; FCCG, 2007; NCCN, 2007; SBHW, 2007; NICE, 2008); three provided no information on the composition of the group (BCCA, 2001; ACR, 2005; ACB, 2007). Five guidelines involved patients, or their representatives or carers in the guideline development group, focus groups or in reviewing draft recommendations (COIN, 1999; NICE, 2002; OMHLTC, 2002; CBO, 2007; NICE, 2008); 13 gave no indication of having incorporated patients’ views or preferences. None of the guidelines were piloted in clinical practice before publication.

A third of the guidelines reported the sources searched to identify the supporting evidence (COIN, 1999; NICE, 2002; SOR, 2006; ACB, 2007; CBO, 2007; NICE, 2008). The quality of the evidence and the strength of the recommendations were graded using a wide variety of schemes. Most of the guidelines mentioned consensus in formulating the recommendations, yet none of them reported formal consensus procedures or fully described the methods used to reach final decisions or resolve disagreements. Only six provided clear and explicit links between the supporting evidence – or its absence – and the recommendations (AUA, 2000; SOR, 2006; CBO, 2007; FCCG, 2007; SBHW, 2007; NICE, 2008). Seven appeared not to have undergone external review before publication (ESTRO, 2000; BCCA, 2001; OMHLTC, 2002; ACB, 2007; EAU, 2007; NCCN, 2007; SBHW, 2007).

Ten of the guidelines had tools for application, as far as we could determine. In most cases, it was a single tool, such as a summary document or patient information. The recent UK guidelines were a notable exception, published together with a short version, quick reference guide and a patient information booklet (NICE, 2008).

The guidelines largely did not consider potential organisational barriers or the cost implications of applying the recommendations, or present review criteria for monitoring and audit. A conflict of interest statement of the guideline group members was missing from 11 of the 18 guidelines reviewed, and only one stated explicitly that the views and interests of the funding body did not influence the recommendations (CCNS, 2006).

## Recommendations on follow-up

### Service organisation

Most of the included guidelines did not address service organisation. The latest UK guidelines on prostate cancer (NICE, 2008) refer to the urological cancer services guidance (NICE, 2002) as the core model for service delivery, which stresses the importance of multidisciplinary team management. In all, nine of the guidelines mentioned the follow-up provider (who) or the setting (where). The UK guidelines show a shift over time from recommending that follow-up after treatment with curative intent takes place in a specialist unit (COIN, 1999) to offering appropriate patients follow-up outside hospital either by a specialist nurse or in primary care (NICE, 2002; NICE, 2008). Recent Dutch guidelines similarly recommend that a specialist nurse or GP can monitor PSA once it is stable (CBO, 2007), but do not require patients to be stable for 2 years before being given this option as stipulated in the UK guidelines (NICE, 2008). Contemporary Swedish and Finnish guidelines have divergent views: the former omitted the GP in recommending follow-up by a urologist, oncologist or specialist nurse, whereas in Finland, only the first post-treatment visit (after prostatectomy, EBRT or brachytherapy) takes place in secondary care with all subsequent follow-up in primary care (FCCG, 2007; SBHW, 2007). Recent guidelines issued in North America continue to recommend that patients are followed up by specialist clinicians after radical treatments (ACR, 2005; CCNS, 2008), although it was one Canadian guideline groups’ policy to refer patients back to the community ‘as far as practicable’ (BCCA, 2001).

Swedish guidelines specified that the urologist does active surveillance, whereas a specialist nurse or a GP can do watchful waiting (SBHW, 2007). In UK guidelines, watchful waiting should normally be provided in primary care, and primary care services have responsibility for the day-to-day management of men with metastatic disease (NICE, 2008). Similarly, according to Finnish guidelines, men undergoing hormone treatment receive all follow-up in primary care (FCCG, 2007).

### Use of tests and examinations

*Prostate-specific antigen testing:* International guidelines agree on the fundamental role of PSA testing in prostate cancer follow-up, but recommendations on the frequency of tests and the duration of follow-up are highly inconsistent (Table 2). The recommended interval between PSA tests in the first year following prostatectomy or radical radiotherapy varies between 3 and 12 months. There is marked variation in the recommended frequency of routine testing relative to duration beyond the first year, irrespective of the type of treatment. The guidelines reflect the lack of consensus on the role of PSA following brachytherapy; those that do recommend regular testing differ in how often and for how long. Guidelines on active surveillance recommend a PSA test every 3–6 months, some increase the interval to 6 months only after the first 2 years (BCCA, 2001; NICE, 2008) or simply advise regular testing (AUA, 2000; NICE, 2002).

UK guidelines advise that men in watchful waiting should have at least one PSA test a year (NICE, 2008) in contrast to the specific (but different) test schedules in Swedish and French guidelines (AFU, 2005; SBHW, 2007). Overall, six guidelines contain similar recommendations on PSA test frequency after the initiation of hormone therapy, but three note that follow-up may need to be tailored to the needs of the patient depending on the type of hormone treatment, symptoms, clinical condition, age and prognosis (CCNS, 2006; ACB, 2007; EAU, 2007). Swedish guidelines recommend more frequent PSA tests for patients with metastases than for those without known metastases (SBHW, 2007) in contrast to one Canadian guideline that recommends routine PSA testing for advanced disease but not for metastatic disease (ACB, 2007).

There is also a high degree of variability between guidelines on what defines biochemical failure, that is, the change in PSA that should prompt further investigation (Supplementary Table 4). Most guidelines adopt expert panel standard definitions for biochemical failure following EBRT, but show a clear lack of consensus in regard to brachytherapy and prostatectomy – and a dearth of advice on active surveillance, watchful waiting and advanced disease.

*Digital rectal examination and other tests:* Guidelines on routine digital rectal examination (DRE) following treatment with curative intent fall into three categories: either it is not recommended (as long as PSA is stable) (CBO, 2007; NICE, 2008) or it is recommended supplementary to PSA testing, either with each PSA test (BCCA, 2001; OMHLTC, 2002; SOR, 2006; EAU, 2007) or less frequently (AFU, 2005; CCNS, 2006; ACB, 2007; NCCN, 2007) (Table 3). The type of curative treatment, prostatectomy or radiotherapy, does not explain these conflicting recommendations. It is also evident that there is no consensus on the use of DRE following brachytherapy. Six guidelines recommend DRE in the course of active surveillance (AUA, 2000; NICE, 2002; OMHLTC, 2002; CCNS, 2006; NCCN, 2007; SBHW, 2007) in contrast to the NICE guidelines’ recommendation against it as long as PSA remains at baseline levels (NICE, 2008). The NICE guidelines also recommend against routine DRE for men undergoing watchful waiting (NICE, 2008). Guidelines that advised on DRE during routine follow-up after initiation of hormone therapy recommend that it should be performed with each PSA test (OMHLTC, 2002; EAU, 2007; NCCN, 2007).

Biopsy, and imaging and biochemical tests other than PSA feature irregularly in follow-up recommendations. Four European guidelines agree that routine biopsy and imaging is unnecessary following treatment with curative intent if patients are asymptomatic and PSA is low and stable (AFU, 2005; SOR, 2006; CBO, 2007; EAU, 2007). Active surveillance should include at least one re-biopsy according to UK guidelines that do not specify the timing (NICE, 2008); other guidelines recommend it within 18 months, then periodically (CCNS, 2006; NCCN, 2007) or every 3 years (BCCA, 2001). Dutch and French guidelines (AFU, 2005; CBO, 2007) do not recommend routine biochemistry, such as creatinine, transaminases and testosterone, for follow-up of asymptomatic patients following treatment with curative intent, but Swedish guidelines do include creatinine and haemoglobin in routine follow-up after curative radiotherapy as well as during active surveillance and watchful waiting (SBHW, 2007). Regular monitoring of creatinine, haemoglobin and liver function tests (alkaline phosphatase or alanine aminotransferase) is recommended in European Association of Urology guidelines on metastatic disease and in concurrent Swedish guidelines on follow-up after hormone therapy (EAU, 2007; SBHW, 2007).

### *Complications and adverse effects*

Specific recommendations on the evaluation of complications and treatment-related adverse effects appear infrequently in guidelines: those for Nova Scotia singularly recommend establishing a specific schedule of follow-up visits after radical treatment to discuss and manage urinary incontinence, erectile dysfunction and sexual health, and suggest routine screening for men considered to be at high risk for psychosocial distress throughout the course of the disease (CCNS, 2006). UK guidelines recommend that men and their partners are given the opportunity to discuss psychosexual problems (NICE, 2008) and that counselling on sexual problems and incontinence is made available for as long as it is needed (NICE, 2002).

## Discussion

This study is the first to review prostate cancer guidelines systematically and to summarise international guideline recommendations on follow-up. Although monitoring PSA remains the cornerstone of follow-up for men with prostate cancer, the diversity of guideline recommendations on the frequency and duration of PSA testing, and components of follow-up other than PSA testing, reflects the current lack of research evidence on which to base firm conclusions. The guidelines provide only broad frameworks for evaluating potential intervention models of follow-up care, particularly in terms of the setting and the composition of the health-care team.

In conducting this review, we had a specific interest in the role of primary care in prostate cancer follow-up. The included guidelines illustrate the disagreement that persists on the extent to which primary care should be involved. The recommendations in recent guidelines that highlight primary-care-based follow-up would require considerable effort and investment to implement – in terms of education, protocols and strategies to change established practice (Grol *et al*, 1998; Foy *et al*, 2002). Furthermore, cancer follow-up in the community requires a close cooperation between primary and secondary care services. However, there has been little research on the best way to manage processes of care involving related actions and decisions by different care providers and, as this review shows, practice guidelines seldom address implementation (Grol *et al*, 2003). Guidelines recommending follow-up in the community give only general guidance, such as agreeing shared care protocols, maintaining close contact between all professionals involved and having mechanisms in place to allow primary care providers access to specialist services. With the current focus on integrated models of chronic care, there are, again, limitations on the usefulness of existing guidelines (Barr *et al*, 2003).

The paucity of high-quality studies in the literature on prostate cancer follow-up has important implications, particularly for primary care, because recommendations based on explicit and non-conflicting scientific evidence are adhered to more in general practice (Grol *et al*, 1998). It underlines the importance of strengthening the evidence base on prostate cancer follow-up and keeping guidelines up to date as new evidence emerges.

Our review has some limitations to consider. Identifying guidelines largely through electronic sources may have introduced bias towards English language guidelines and guidelines produced by larger, well-established organisations. Conversely, searching multiple sources, using foreign language search terms and pre-defining inclusion criteria on guideline developers, should have reduced the risk of language bias and failing to identify guidelines from eligible sources. We acknowledge that all relevant information may not have been included in the translations of foreign language guidelines and that quality assessment based on translations may not be entirely accurate. Nevertheless, our findings are consistent with earlier studies that have noted conflicting guideline recommendations on prostate cancer diagnosis and treatment (Meyer *et al*, 2006) and shortcomings in the methods used by leading urological associations to develop guidelines for prostate cancer (Dahm *et al*, 2007). The guidelines we reviewed were produced in developed countries with similar prostate cancer incidence trends and management options, consequently our findings may not be generalisable to other settings.

The methodological quality of the guidelines included in our review was heterogeneous and in most cases moderate to poor. We used the AGREE instrument as the indicator of guideline quality because it is validated and considered to be the international standard in guideline assessment (Vlayen *et al*, 2005). The criteria mainly address the methods of guideline development and the quality of reporting. The high-quality guidelines in this review were generally well reported and achieved high scores on almost all domains. However, inadequate and incomplete reporting cannot be ruled out as a reason for lower quality scores (Fervers *et al*, 2005). Guidelines of similar methodological quality still differ in their recommendations on prostate cancer follow-up, indicating that important influences on guideline development are not always explicit. Furthermore, in common with other critical appraisal tools for guidelines, the AGREE instrument does not assess the clinical content of the recommendations or the quality of the supporting evidence. Good methodological quality does not necessarily indicate good-quality recommendations (Burgers, 2006).

Most of the guidelines in our review combined an evidence-based approach with informal consensus – reflecting the status of the international literature in this field (Martin *et al*, 2006; Warren and McFarlane, 2007). When recommendations are formulated by consensus comprehensive stakeholder involvement, transparent consensus procedures and editorial independence are especially important for guideline credibility (Fervers *et al*, 2005). We found that these important elements are often not evident in prostate cancer guidelines. The balance between individual and speciality biases in guidelines development groups could, in part, account for different recommendations on who provides follow-up and in what setting. For example, guidelines recommending that clinical specialists provide follow-up were more likely to have been developed exclusively by representatives of that group. Those that recommend involvement of nurse specialists and primary care were largely developed by multidisciplinary groups that included a range of care providers and sought the views of service users. Research in other clinical areas has shown that speciality groups favour procedures in which they have a vested interest and even when presented with the same evidence will reach different conclusions than wider multidisciplinary groups (Murphy *et al*, 1998; Shekelle *et al*, 1999; Fretheim *et al*, 2006). Members of guideline development groups may also more readily endorse models of care that have already been implemented in their locality. When the consensus procedure is ill defined, it is difficult to ascertain which aspects might have influenced the outcome or how reliably expert opinion and stakeholders’ preferences were incorporated (Grol *et al*, 2003; Raine *et al*, 2005).

Reviews of international guidelines such as ours often find that recommendations are shaped or constrained by the structure and organisation of their country's health-care systems, even when the evidence is incontrovertible (Eisinger *et al*, 1999; Burgers *et al*, 2002; Philip *et al*, 2003). In the United Kingdom, for example, cancer plans (NHS, 2000; Department of Health, 2007) have emphasised patient preference on the delivery of cancer care and alternative models of health-care delivery, which bring care closer to the patient – these elements are reflected in the latest UK guidelines on prostate cancer (NICE, 2008).

Ultimately, the usefulness of a guideline needs to take account of the impact on patient outcomes of applying the recommendations in local settings. Systematic appraisal can aid informed judgment on guideline quality: guideline recommendations can only be rigorously tested by incorporating them in the development of interventions for evaluation in randomised trials.

## Conclusions

This review shows the current status of international guidelines in prostate cancer follow-up. The variability in recommendations to some extent reflects the lack of definitive evidence from research in this field. Choices over management in prostate cancer, including follow-up, can only be informed to a limited extent by evidence from high-quality trials.

The review illustrates the importance of rigorous and transparent guideline development processes when research evidence is limited, without which it is difficult to assess factors such as independence and impartiality. It also highlights the need for robust primary research to improve the evidence base for prostate cancer follow-up – particularly research to inform best practice models of care. Only then can the many new models of care emerging internationally be shaped in a rational, evidence-based way.

## Figures and Tables

**Table 1:** Included guidelines

**Guideline**	**Title**	**Source**	**Quality** a
NICE (2008)	Prostate cancer: diagnosis and treatment	National Institute for Health and Clinical Excellence	High
EAU (2007)	Guidelines on prostate cancer	European Association of Urology	Low
CBO (2007)	Guideline on prostate cancer: diagnosis and treatment [Dutch]	Dutch Institute for Healthcare Improvement (CBO)	High
NCCN (2007)	Prostate cancer: Clinical Practice Guidelines in Oncology	The National Comprehensive Cancer Network	Low
ACB (2007)	Clinical Guidelines: Prostate cancer	Alberta Cancer Board	Low
FCCG (2007)	Prostate cancer: Current Care Guidelines [Finnish]	Finnish Medical Society Duodecim	High
SBHW (2007)	National guideline for prostate cancer management [Swedish]	Swedish National Board of Health and Welfare	Moderate
ESMO (2006)	Prostate cancer: clinical recommendations for diagnosis, treatment and follow-up	European Society for Medical Oncology	Low
SOR (2006)	Standards, Options and Recommendations for the management of non-metastatic prostate cancer [French]	French Federation of Comprehensive Cancer Centres and French Urological Association	Moderate
CCNS (2006)	Guidelines for the management of prostate cancer	Cancer Care Nova Scotia	Moderate
AFU (2005)	Follow-up of prostate cancer [French]	French Urological Association	Low
ACR (2005)	Practice guideline for transperineal permanent brachytherapy of prostate cancer	American College of Radiology	Moderate
OMHLTC (2002)	PSA Clinical Guidelines	Ontario Ministry of Health and Long-Term Care	Moderate
NICE (2002)	Improving Outcomes in Urological Cancers	National Institute for Health and Clinical Excellence	High
BCCA (2001)	Cancer Management Guidelines: Prostate	British Colombia Cancer Agency	Low
ESTRO (2000)	Recommendations on permanent seed implantation for localised prostate cancer	European Society for Therapeutic Radiology and Oncology	Low
AUA (2000)	Prostate-specific antigen (PSA) best practice policy	American Urological Association	Moderate
COIN (1999)	Guidelines on the management of prostate cancer	Royal College of Radiology, British Association of Urological Surgeons	Moderate

Abbreviations: ACB=Alberta Cancer Board; ACR=American College of Radiology; AFU=French Urological Association; AUA=American Urological Association; BCCA=British Colombia Cancer Agency; CBO=Dutch Institute for Healthcare Improvement; CCNS=Cancer Care Nova Scotia; COIN=Royal College of Radiology, British Association of Urological Surgeons; EAU=European Association of Urology; ESMO=European Society for Medical Oncology; ESTRO=European Society for Therapeutic Radiology and Oncology; FCCG=Finnish Current Care Guidelines; NICE=National Institute for Health and Clinical Excellence; NCCN=National Comprehensive Cancer Network; OHMLTC=Ontario Ministry of Health and Long-Term Care; SBHW=Swedish National Board of Health and Welfare; SOR=Standards, Options and Recommendations.

a Quality was assessed using the Appraisal of Guidelines Research and Evaluation instrument (www.agreecollaboration.org).

**Table 2:** Guidelines follow-up recommendations on PSA testing

**Guideline**	**Treatment with curative intent**	**Prostatectomy**	**External beam radiotherapy**	**Brachytherapy**	**Active surveillance**	**Watchful waiting**	**Advanced and metastatic disease**
NICE (2008)	6 weeks post-treatment, at least every 6 months for the first 2 years, then at least annually				Every 3 months in the first 2 years, then 6 monthly	At least once a year	
EAU	At 3, 6 and 12 months, then every 6 months until 3 years, then annually						3 and 6 months after initiating treatment, then every 3–6 months for M1 disease and good treatment response
CBO	At 6 weeks, 3, 6, 9 and 12 months, then every 6–12 months for 5–10 years						
FCCG		6–12 months after surgery, then every 6 months for 5 years, then every 12 months	At 3 and 12 months after treatment, then every 6–12 months for up to 5 years, then annually	At 3 months after treatment, then every 6–12 months for up to 5 years, then annually			Every 3–6 months for 5 years, then every 12 months for men on hormone therapy
SBHW	Every 6 months for 2–5 years				Every 3–6 months	Every 6–12 months	Every 6–12 months for patients without known metastases; every 3–6 months for patients with metastases; at least every 3 months for patients with clinical progression
NCCN	Every 6–12 months for 5 years, then annually				Every 6 months if life expectancy ⩾10 years, every 6–12 months if <10 years		Every 3–6 months after initial therapy for N1 or M1 disease
ACB		4–8 weeks after surgery, then every 6 months for 2 years, then annually	Every 6 months for 2 years, then annually (intermediate risk)	Every 6 months for 2 years, then annually (intermediate risk)	As a further management option following radical prostatectomy: PSA every 3–4 months		Every 6 months for advanced disease if it will affect management
		Low risk may have PSA only annually					PSA should not be done routinely for metastatic disease, only when it will affect management
ESMO		PSA should be monitored					
SOR		Between 1 and 3 months, then every 3 months in the first year (less if < limit of detection) and every 6 months for the next 7 years	Every 6 months for an indefinite period	At regular intervals			
CCNS		Every 3–12 months in years 1–3 and every 6–12 months from year 3 onwards	Every 3–4 months in years 1–5, then every 3–6 months beyond 5 years		Every 6 months		
AFU		Within 3 months, then at 6 months, then, every 6 months for 3 years, then annually	Every 6 months for 3 years, then annually	Every 6 months for 10 years is customary practice	Every 3–6 months	Every 6 months for 4 years, then annually	At 3 months to determine nadir following hormone therapy
ACR				Follow-up at 3–6-month intervals for 1–2 years, then periodically, may include PSA			
OMHLTC		At 3–12-month intervals	At 3–12-month intervals	Role not yet established	At 3–6-month intervals		At 3–6-month intervals for men undergoing hormone therapy
NICE (2002)					Regular		
BCCA		Every 3 months in the first year, then every 6 months	Every 6 months for 3 years, then annually		Every 3 months for 2 years, then 6 monthly		
ESTRO				Follow-up every 3 months for the first year, then every 6 months to 5 years, then annually, should include PSA			
AUA		Periodic	Periodic, no more than every 3–6 months		Consider regular tests		
COIN							It is sensible to monitor PSA every 3 months when hormone treatment for metastatic disease is deferred

Abbreviations: DRE=digital rectal examination; PSA=prostate-specific antigen.

**Table 3:** Guidelines follow-up recommendations on DRE

**Guideline**	**Treatment with curative intent**	**Prostatectomy**	**External beam radiotherapy**	**Brachytherapy**	**Active surveillance**	**Watchful waiting**	**Advanced and metastatic disease**
NICE (2008)	Not recommended as routine while PSA remains at baseline levels				Not recommended while PSA remains at baseline levels	Not recommended while PSA remains at baseline levels	-
EAU	At 3, 6 and 12 months, then every 6 months until 3 years, then annually						At 3 and 6 months, then every 6 months for M0 and good treatment response, every 3–6 months for M1 and good treatment response
CBO	Not recommended as routine if PSA is decreasing or low and stable						
SBHW					Every 3–6 months		
NCCN	Annually				Every 6 months if life expectancy 10 years, every 6–12 months if <10 years		Every 3–6 months after initial therapy for N1 or M1
ACB	Annually			Annually			
SOR		Optional for patients with total serum PSA < limit of detection	Every 6 months for an indefinite period	At regular intervals			
CCNS			Every 6–24 months in years 1–5, then every 1–3 years		Every 6 months		
AFU		Recommended if PSA detectable or indicates a higher grade tumour or risk of local relapse is important	Annually	Annually for 10 years is customary practice			
ACR				Follow-up at 3–6 month intervals for 1–2 years, then periodically, may include DRE			
OMHLTC		At 3–12-month intervals	At 3–12-month intervals		At 3–6-month intervals		At 3–6-month intervals for men undergoing hormone therapy
NICE (2002)					Regular		
BCCA		Every 3 months in the first year, then every 6 months			Every 6 months for 2 years, then 6 monthly		
ESTRO				Follow-up every 3 months for the first year, then every 6 months to 5 years, then annually, should include DRE			
AUA					Consider regular tests		

Abbreviations: DRE=digital rectal examination.
