# Supplementary material for: Follow-up care for men with prostate cancer and the role of primary care: a systematic review of international guidelines
Source: Br J Cancer. 2009 May 12;100(12):1852–60. doi: 10.1038/sj.bjc.6605080 (PMC2714251; doi:10.1038/sj.bjc.6605080)
Supplement: Supplementary Table 1 [file 6605080x1.doc]

Supplementary Table 1. Sources searched to identify relevant guidelines

| **Source and strategy** |
| --- |
| SUMSearch:  guideline* AND prostatic neoplasms [MeSH]  practice guideline* AND prostatic neoplasms [MeSH]  recommendation* AND prostatic neoplasms [MeSH]  standard* AND prostatic neoplasms [MeSH]  Searches restricted to “Practice Guidelines” (NGC and PubMed); SUMSearch links used to search for guidelines at PubMed |
| TRIP database, US National Guideline Clearinghouse, German Clearinghouse for Guidelines, *e*Guidelines, Medical Journal of Australia Clinical Guidelines, NLH Cancer Specialist Library, NHS National Library for Health, UKMiCentral, CMA Infobase, The Complete Urology and Andrology Info Base, Health Services/Technology Assessment Text, Clinical Knowledge Summaries |
| Websites of professional societies, government-supported agencies, non-government funded organisations searched individually:   - National Institute for Health and Clinical excellence (NICE) - Scottish Intercollegiate Guidelines Network (SIGN) - British Association of Urological Surgeons (BAUS) - British Association of Urological Nurses (BAUN) - British Prostate Group (BPG) - Royal College of Radiologists (RCR) - Royal College of General Practitioners (RCGP) - European Association of Urology (EAU) - European Society for Medical Oncology (ESMO) - l'Association Francaise d'Urologie (AFU) - Dutch association of comprehensive cancer centres (ACCC) - Dutch College of General Practitioners (NHG) - Dutch Institute for Healthcare Improvement (CBO) - Association of the Scientific Medical Societies in Germany (AWMF) - Programm für Nationale VersorgungsLeitlinien - Danish Secretariat for Clinical Guidelines (DSCG) - Scandinavian Association of Urology (NUF) - Swedish Council on Technology Assessment in Health Care (SBU) - Swedish Board for Health and Welfare (Socialstyrelsen) - Sociedad Vasca de Medicina Familiar y Comunitaria (OSATZEN) - Sistema Nazionale Linee Guida – ISS, - Agency for Healthcare Research and Quality (AHRQ) - National Comprehensive Cancer Network (NCCN) - American Society for Clinical Oncology (ASCO) - American College of Radiology (ACR) - American Urological Association (AUA) - American Society for Therapeutic Radiology and Oncology (ASTRO) - Canadian Urological Association (CUA) - Canadian Medical Association (CMA) - Cancer Care Ontario - British Colombia Cancer Agency (BCCA) - Cancer Care Nova Scotia (CCNS) - Alberta Clinical Practice Guidelines - Ontario Ministry of Health and Long-term Care - National Health and Medical Research Council (NHMRC) - New Zealand Guidelines Group (NZGG) |
| Ovid MEDLINE(R) 1950 to August Week 5 2007  Search Strategy 1  1 prostatic neoplasms/  2 (prostate adj3 cancer).ti,ab.  3 (urologic$ adj2 cancer).ti,ab.  4 1 or 2 or 3  5 ((guideline$ or guidance) not screening).ti.  6 4 and 5  Search Strategy 2  1 prostatic neoplasms/  2 (prostate adj3 cancer).ti,ab.  3 (urologic$ adj2 cancer).ti,ab.  4 1 or 2 or 3  5 limit 4 to guideline  6 limit 4 to practice guideline  7 5 or 6  EMBASE 1980 to 2007 Week 35  1 prostatic neoplasms/  2 (prostate adj3 cancer).ti,ab.  3 (urologic$ adj2 cancer).ti,ab.  4 1 or 2 or 3 (34183)  5 ((guideline$ or guidance) not screening).ti.  6 4 and 5 |
