# Supplementary material for: Follow-up care for men with prostate cancer and the role of primary care: a systematic review of international guidelines
Source: Br J Cancer. 2009 May 12;100(12):1852–60. doi: 10.1038/sj.bjc.6605080 (PMC2714251; doi:10.1038/sj.bjc.6605080)
Supplement: Supplementary Table 2 [file 6605080x2.doc]

Supplementary Table 2. Excluded guidelines

| **Title, Source, Date** | **Reason for exclusion** |
| --- | --- |
| Guideline for the management of clinically localized prostate cancer. American Urological Association 2007 | Mentions follow up but has no recommendations on the process; has recommendations on watchful waiting and active surveillance as management options (and a ‘Standard’ statement that follow up should be tailored according to the aim of 2^nd^-line therapy for patients choosing active surveillance) but does not contain recommendations on the process |
| Initial hormonal management of androgen-sensitive metastatic, recurrent, or progressive prostate cancer. ASCO 2007 | Recommends that patients who decide to wait until symptoms develop should have regular visits for monitoring, but no details on the process. |
| **Advice on the development of low dose rate (permanent seed implant) brachytherapy services for localised prostate cancer in England. Department of Health UK 2006** | Describes follow up as a part of the procedure but does not contain recommendations on follow up |
| Guideline for the use of PSA and the early diagnosis of prostate cancer. Alberta Clinical Practice Guidelines (Towards Optimized Practice) 2006 | References another guideline for recommendations on PSA for follow up of prostate cancer (see included guideline ACB 2007) |
| Guidelines for primary radiotherapy of patients with prostate cancer. EORTC 2006 | Does not mention follow up |
| Interdisciplinary recommendations concerning the therapy for hormone-refractory prostatic carcinoma. 2006 | Includes monitoring of docetaxel therapy, not follow up |
| Locally advanced (high-risk) prostate cancer. American College of Radiology 2006 | Appropriateness criteria for radiotherapy, does not mention follow up |
| Postradical prostatectomy irradiation in prostate cancer. American College of Radiology 2006 | Appropriateness criteria for radiotherapy, does not mention follow up |
| MDT (Multi-disciplinary team) guidance for managing prostate cancer. BAUS, British Uro-oncology Group (BUG), British Prostate Group (BPG) 2005 | Does not mention follow up; has recommendations on watchful waiting and active surveillance as management options, gives patient selection characteristics and an overview of, but not recommendations on, the process |
| Post-treatment follow up of prostate cancer. American College of Radiology 2005 | Appropriateness criteria for radiologic procedures for post treatment follow up once recurrence or progression is suspected |
| Position statement: redefining the management of hormone-refractory prostate carcinoma. Society of Urologic Oncology 2005 | Does not mention follow up |
| Non-hormonal systemic therapy in men with metastatic hormone-refractory prostate cancer: A clinical practice guideline. Program in Evidence-based Care, Cancer Care Ontario Genitourinary Cancer Disease Site Group 2005 | Does not mention follow up |
| Cáncer de próstata de alto riesgo y diseminado: Documento de Consenso de Recomendaciones. Grupo de Expertos en Cáncer de Próstata 2005 | Expert group consensus opinion; not clearly identified as the product of an eligible source |
| Prostate cancer management. l'Association Francaise d'Urologie 2004 | Does not contain recommendations on follow up; mentions surveillance as a management option but does not contain recommendations on the process |
| Management of prostate-specific antigen relapse in prostate cancer. European Consensus 2004 | Gives definitions for relapse but contains no recommendations on follow up |
| Management of locally advanced prostate cancer: a European consensus. 2003 | Does not mention follow up |
| Prostate cancer management clinical guidelines. Royal College of Surgeons in Ireland 2002 | Mentions follow up but does not contain recommendations on the process; has a recommendation on surveillance as a management option, but not the process  Contains these Quality Standards:  Patients [post prostatectomy] should be followed in a specialised urology department.  Patients undergoing radical radiotherapy should be followed by a specialist unit. |
| Clinical Practice Guidelines: Evidence–based information and recommendations for the management of localised prostate cancer. National Health and Medical Research Council (NHMRC) Australia 2002 | Does not mention follow up; describes deferred treatment as a management option but does not contain recommendations on the process |
| Leitlinien zur therapie von prostatakarzinomen. Deutschen Krebsgesellschaft 1999 | Does not mention follow up; watchful waiting/monitoring mentioned as management options but no recommendations on the process |
| Consensus statement: guidelines for PSA following radiation therapy. ASTRO 1997 | Recommendations on benchmarks for PSA failure only |
| Prostate disease: management options for the primary healthcare team. BPG 1995 | States that practice guidelines could not be specified; describes general guidance on monitoring in primary care |
| Guidelines for the cancer-related checkup: prostate and endometrial cancers. American Cancer Society 1993 | Concerns early detection of prostate cancer in asymptomatic men (subsequently updated annually as guidelines for the early detection of prostate cancer) |
