# Supplementary material for: Follow-up care for men with prostate cancer and the role of primary care: a systematic review of international guidelines
Source: Br J Cancer. 2009 May 12;100(12):1852–60. doi: 10.1038/sj.bjc.6605080 (PMC2714251; doi:10.1038/sj.bjc.6605080)
Supplement: Supplementary Table 3 [file 6605080x3.doc]

**Supplementary Table 3. Included guidelines Appraisal of Guidelines Research and Evaluation (AGREE) domain scores**

| ***DOMAIN*** | ***NICE 2008*** | ***EAU*** | ***CBO*** | ***NCCN*** | ***ACB*** | ***FCCG*** | ***SBHW*** | ***ESMO*** | ***SOR*** | ***CCNS*** | ***AFU*** | ***ACR*** | ***OMH-LTC*** | ***NICE 2002*** | ***BCCA*** | ***ESTRO*** | ***AUA*** | COIN |
| --- | --- | --- | --- | --- | --- | --- | --- | --- | --- | --- | --- | --- | --- | --- | --- | --- | --- | --- |
| Scope and purpose | 100 | 11 | 89 | 11 | 33 | 61 | 44 | 11 | 56 | 22 | 11 | 33 | 44 | 66 | 11 | 33 | 56 | 22 |
| Stakeholder involvement | 75 | 8 | 66 | 25 | 8 | 46 | 42 | 25 | 25 | 33 | 8 | 33 | 66 | 75 | 8 | 33 | 42 | 66 |
| Rigour of development | 95 | 28 | 86 | 33 | 19 | 86 | 52 | 19 | 76 | 38 | 24 | 33 | 10 | 66 | 10 | 24 | 38 | 52 |
| Clarity and presentation | 100 | 92 | 76 | 92 | 66 | 96 | 83 | 92 | 92 | 92 | 75 | 66 | 50 | 100 | 42 | 66 | 42 | 100 |
| Applicability | 33 | 0 | 56 | 0 | 0 | 17 | 56 | 0 | 0 | 0 | 0 | 0 | 11 | 88 | 0 | 11 | 0 | 33 |
| Editorial independence | 66 | 0 | 83 | 50 | 0 | 92 | 0 | 0 | 33 | 83 | 0 | 0 | 0 | 0 | 0 | 0 | 50 | 33 |
|  |  |  |  |  |  |  |  |  |  |  |  |  |  |  |  |  |  |  |

Domain Score = (actual score − minimum possible score) ∕ (maximum possible score − minimum possible score)

Overall quality: High – the guideline rated high (3 or 4) on the majority of items and most domain scores are >60%; Moderate – the guideline rated high (3 or 4) or low (1 or 2) on a similar number of items and most domain scores are between 30 and 60%; Low – the guideline rated low (1 or 2) on the majority of items and most domain scores are <30%
