# Supplementary material for: Follow-up care for men with prostate cancer and the role of primary care: a systematic review of international guidelines
Source: Br J Cancer. 2009 May 12;100(12):1852–60. doi: 10.1038/sj.bjc.6605080 (PMC2714251; doi:10.1038/sj.bjc.6605080)
Supplement: Supplementary Table 4 [file 6605080x4.doc]

**Supplementary Table 4. Included g**uidelines definitions of biochemical failure

| **Guideline** | **Prostatectomy** | **External beam radiotherapy** | **Brachytherapy** | **Active surveillance** | **Watchful waiting** | **Advanced and metastatic disease** |
| --- | --- | --- | --- | --- | --- | --- |
| NICE 2008 | PSA >0.4ng/ml and rising—residual disease that may lead to clinical progression | PSA rising >2ng/ml above nadir^1^ | PSA rising >2ng/ml above nadir^1^ | A rise in PSA is evidence of disease progression | Rapidly rising PSA is evidence of significant disease progression |  |
| EAU | 2 sequential PSA values ≥0.2ng/ml | PSA rising >2ng/ml above nadir^1^ |  |  |  | Hormone therapy: rising PSA usually precedes onset of clinical symptoms |
| CBO | 2 sequential PSA values >0.2ng/ml, test interval 2-3 mo | 3 sequential rises in PSA^2^, min interval 3 mo | 3 sequential rises in PSA^2^, min 3 mo interval |  |  |  |
| FCCG | Refer to secondary care if 2 sequential rises in PSA in 3 mo | Refer to secondary care if 2 sequential rises in PSA in 3 mo |  |  |  | Hormone therapy: 2 sequential rises in PSA in 3 mo—consult urologist |
| NCCN | PSA rising on 2 or more sequential tests | PSA rising >2ng/ml above nadir^1^ |  | Criteria for progression not well defined, requires physician judgment; may have occurred if PSA dt <3yrs or velocity >0.75 |  | Disseminated disease: 3 sequential rises in PSA^2^ |
| ACB |  |  | Radical therapy may be initiated if PSA dt is short |  |  |  |
| SOR | 2 sequential PSA values >0.1ng/ml | 3 sequential rises in PSA^2^, min interval 3 mo | 3 sequential rises in PSA^2^, 3 mo interval |  |  |  |
| CCNS | If PSA rises to >0.2ng/ml on 2 sequential tests consider salvage therapy | If 3 consecutive rises in PSA 3-4 mo apart and PSA ≥1.5ng/ml consider salvage therapy |  |  |  | ADT: rising PSA indicator of change in management |
| AFU | 2 or 3 sequential PSA values >0.1ng/ml | 3 sequential rises in PSA^2^ at 1 mo intervals | ASTRO^2^ criteria difficult to apply in the first years because of PSA rebound |  |  |  |
| ACR |  |  | Best definition of biochemical failure not yet determined |  |  |  |
| BCCA | 2 successive rises in PSA to >0.3ng/ml | At least 2 sequential rises in PSA at least 1 mo apart to at least 1.5ng/ml^3^ | May experience a bounce typically 1-3 yrs post treatment, seek advice from a radiation oncologist | PSA dt <3 yrs based on a minimum of 3 values |  |  |
| ESTRO |  |  | 3 sequential rises in PSA |  |  |  |
| AUA | Detectable PSA indicates recurrence | 3 sequential rises in PSA^2^, min interval 3-6 mo or nadir >0.5ng/ml |  |  |  |  |
| COIN | Mentions ASTRO 1997 definition of biochemical relapse: 3 sequential rises in PSA 3-4 mo apart in 1^st^ yrs, 6 mo thereafter |  |  |  | In first line management of metastatic disease a rapid rise in PSA is probably an indication to commence treatment |  |

^1^ Roach M, Hanks G, Thames H, *et al* (2005) Defining biochemical failure following radiotherapy with or without hormonal therapy in men with clinically localized prostate cancer: recommendations of the RTOG-ASTRO Phoenix consensus conference. Int J Radiation Oncology Biol Phys **65**:965-974

^2^ American Society for Therapeutic Radiology and Oncology Consensus Panel (1997) Consensus statement: guidelines for PSA following radiation therapy. Int J Radiat Oncol Biol Phys **65**:1035-1041

^3^ Pickles T, Duncan GG, Kim-sing C, *et al* (1999) PSA relapse definitions – the Vancouver rules show superior predictive power. Int J Radiat Oncol Biol Phys **43**:699-700
